# First Occurrence of *Eustrongylides* spp. (Nematoda: Dioctophymatidae) in a Subalpine Lake in Northwest Italy: New Data on Distribution and Host Range

**DOI:** 10.3390/ijerph17114171

**Published:** 2020-06-11

**Authors:** Vasco Menconi, Maria Vittoria Riina, Paolo Pastorino, Davide Mugetti, Serena Canola, Elisabetta Pizzul, Maria Cristina Bona, Alessandro Dondo, Pier Luigi Acutis, Marino Prearo

**Affiliations:** 1 The Veterinary Medical Research Institute for Piemonte, Liguria and Valle d’Aosta, Via Bologna 148, 10154 Torino, Italy; vasco.menconi@izsto.it (V.M.); mariavittoria.riina@izsto.it (M.V.R.); davide.mugetti@izsto.it (D.M.); serena.canola@izsto.it (S.C.); cristina.bona@izsto.it (M.C.B.); alessandro.dondo@izsto.it (A.D.); pierluigi.acutis@izsto.it (P.L.A.); marino.prearo@izsto.it (M.P.); 2 Department of Life Sciences, University of Trieste, via Giorgieri 10, 34127 Trieste, Italy; pizzul@units.it

**Keywords:** public health, zoonoses, epidemiology, fishbone zoonotic parasite

## Abstract

The genus *Eustrongylides* includes nematodes that infect fish species and fish-eating birds inhabiting freshwater ecosystems. Nematodes belonging to the genus *Eustrongylides* are potentially pathogenic for humans; infection occurs after the consumption of raw or undercooked fish. In the two-year period 2019–2020, a total of 292 fish belonging to eight species were examined for the occurrence of *Eustrongylides* spp. from Lake San Michele, a small subalpine lake in northwest Italy. The prevalence of infestation was 18.3% in *Lepomis gibbosus*, 16.7% in *Micropterus salmoides*, and 10% in *Perca fluviatilis*. The other five fish species (*Ameiurus melas*, *Ictalurus punctatus*, *Squalius cephalus*, *Carassius carassius*, and *Scardinius erythrophthalmus*) were all negative for parasite presence. There were no significant differences in prevalence between the three fish species (Fisher’s exact test; *p* = 0.744). The mean intensity of infestation ranged from 1 (*M. salmoides* and *P. fluviatilis*) to 1.15 (*L. gibbosus)*, and the mean abundance ranged from 0.1 (*P. fluviatilis*) to 0.28 (*L. gibbosus*). There were significant differences in the infestation site between the four muscle quadrants (anterior ventral, anterior dorsal, posterior ventral, and posterior dorsal) and the visceral cavity (Kruskal–Wallis test; *p* = 0.0008). The study findings advance our knowledge about the distribution and host range of this parasite in Italy.

## 1. Introduction

The genus *Eustrongylides* Jägerskiöld, 1909 (Family Dioctophymatidae) includes nematodes that infect numerous fish species and fish-eating birds inhabiting freshwater ecosystems. *Eustrongylides* species have a heteroxenous life cycle. Among fish-eating birds, the great cormorant (*Phalacrocorax carbo* Linnaeus, 1758) has been frequently reported to be a definitive host [1,2]. Aquatic oligochaetes (i.e., *Lumbriculus variegatus, Tubifex tubifex,* and *Limnodrilus* spp.) are the first intermediate hosts, while fish, amphibians, and/or reptiles are the second intermediate hosts. Furthermore, predatory fish, amphibians, and reptiles may serve in the life cycle as paratenic hosts [3,4,5]. Though the number of recorded cases is low, humans can enter the cycle as accidental hosts [6].

The geographical distribution of the genus *Eustrongylides* is vast (Asia, Africa, and North and South America) [4,7,8,9,10,11,12,13,14,15] probably due to live fish translocation and broad migratory routes of definitive hosts [16,17]. In Italy, *Eustrongylides* nematodes were first reported in *Perca fluviatilis, Micropterus salmoides*, and *Atherina boyeri* from Lake Trasimeno [15,18,19]. Furthermore, Mazzone et al. [20] first described *E. excisus* adults by morphological and molecular analyses.

Species belonging to the genus *Eustrongylides* are pathogenic for birds, fish, and humans [18,21,22]. In birds, the parasite is usually isolated in the wall of the proventriculus, ventriculus, and intestine, where it forms tunnels and provokes granulomatous inflammatory reaction [23]. In fish, infection may result in massive disease and an evident inflammatory response [18,24]. Larvae are found encapsulated in the muscles or visceral organs or free in the body cavity of fish hosts [25]. Death is relatively rare and generally from secondary infections consequent to a weakened immune system [5]. In humans, eustrongylidosis is a parasitic disease contractable after the consumption of raw/undercooked fish infected by the larval stages of the parasite [26]. Symptoms are generally gastrointestinal (e.g., gastritis or enteritis) [6,27,28] and may progress to intestinal perforation [21]. Eberhard and Ruiz-Tiben [29] described a cutaneous form of *Eustrongylides* in two people from South Sudan. The only effective treatment is surgical removal of larvae [21].

Strict application of inspection regulations of fish intended for human consumption and proper food preparation are the best ways to avoid foodborne parasitic zoonoses. Though no cases of human eustrongylidosis have been recorded in Italy, these nematodes merit attention because of their wide distribution and zoonotic potential. In response to reports from sports fishermen about the presence of “red worms” in fish caught from Lake San Michele (Piedmont, northwest Italy) during 2019 and 2020, a parasitological survey of several fish species was carried out. The main aim of this study was to report the presence of *Eustrongylides* nematodes in a subalpine area and to improve our knowledge about the distribution and host range of this parasite in Italy.

## 2. Materials and Methods 

### 2.1. Study Area

**Figure 1:**
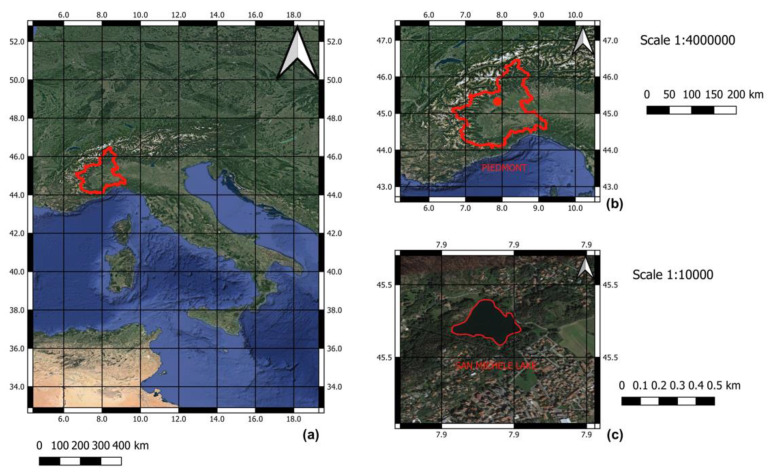
Sampling site: (**a**) Italy with insert, (**b**) Piedmont (outlined in red), and the location of (**c**) Lake San Michele (outlined in red; 45°28′39.1″ N 7°53′18.2″ E).

Lake San Michele (241 m a.s.l.) is one out of a complex of five shallow lakes (Cinque Laghi di Ivrea) in Piedmont (Province of Turin, northwest Italy) (Figure 1).

The lake has a surface area of 0.07 km^2^ and a maximum depth of 19 m [30]. The lake’s biodiversity of fish species and proximity to inhabited centers make it an attractive location for recreational fishing.

### 2.2. Fish Collection and Anatomopathological and Parasitological Examination 

This study was carried out from September 2019 to February 2020. Fish were sampled using multimesh benthic and pelagic gillnets (mesh size, 10 to 55 mm) placed according to the lake’s bathymetry profile, following the standardized method for fish sampling in European lakes [31]. Permission for fish sampling was obtained from the Città Metropolitana di Torino (authorization no. 45-28591/18), as required by local laws. The fish species were black bullhead (*Ameiurus melas*)*,* channel catfish (*Ictalurus* *punctatus*)*,* European chub (*Squalius cephalus*), crucian carp (*Carassius carassius*), pumpkinseed (*Lepomis gibbosus*), largemouth bass (*Micropterus* *salmoides*), European perch (*Perca fluviatilis*), and rudd (*Scardinius erythrophthalmus*). A sample from each species was retained for parasitological analysis. Fish were transported in cold boxes kept at 4 °C to the Fish Diseases Laboratory of the Veterinary Medical Research Institute for Piemonte, Liguria and Valle d’Aosta (Turin, Italy) for analysis. Each fish was weighed (g), measured for total length (cm), and subjected to anatomopathological examination. Skeletal muscle and internal organs were examined for zoonotic helminths. Internal organs were removed from the body cavity and placed in Petri dishes containing saline solution and inspected. Skeletal musculature was fileted in 2–3 mm slices and observed by transillumination (UVP White Light Transilluminators, TW-43, Analytik Jena, Jena, Germany) to detect encysted nematodes. Isolated nematodes were excysted with a fine needle, rinsed in deionized water, and fixed in 96% molecular grade ethanol for molecular analysis. Isolated nematodes were classified at the genus level by morphological examination according to keys provided by Moravec [24]. Nematode location in the skeletal muscle was recorded: anterior ventral (AV), which is the belly flap; anterior dorsal (AD); posterior ventral (PV); and posterior dorsal (PD).

### 2.3. Molecular Analysis

DNA extraction was performed on the entire larvae using a commercial kit (Extractme Genomic DNA kit, Blirt S.A., Gdańsk, Poland). Total DNA was extracted following the manufacturer’s instructions. According to Gustinelli et al. [32], species identification was performed by sequencing the internal transcribed spacer (ITS) that is the conserved regions in samples belonging to the same species. Both strands were sequenced, and they were aligned using Seqman Software (Lasergene). The ITS region was sequenced and compared with similar sequences on the GenBank database using BLASTn. An identity of 98% was considered a cutoff to assign the species.

### 2.4. Statistical Analysis

The Shapiro–Wilk test was used to verify normality of data distribution. The prevalence of infestation was calculated for each species. Differences in the prevalence of infestation between the fish species were tested using the Fisher’s exact test. Mean intensity and mean abundance of infestation were calculated according to Bush [33].

Whereas the Shapiro–Wilk test showed that the data deviated significantly from a normal distribution, the nonparametric Kruskal–Wallis test was used to determine differences in the infestation sites in the musculature (AD, AV, PD, and PV) and the visceral cavity (VC). Dunn’s post hoc test was used for multiple comparisons. Significance was set at 0.05 %. Statistical analyses were performed using open source data analysis software RStudio^®^ version 1.1.463 (RStudio, Inc., Boston, MA, USA).

## 3. Results

**Figure 2:**
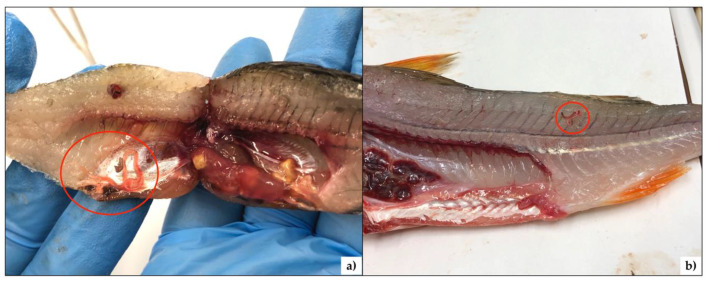
Larvae of *Eustrongylides* spp. (red circles) in (**a**) the visceral cavity of *Lepomis gibbosus* and (**b**) in the posterior dorsal portion of musculature in *Perca fluviatilis*.

A total of 292 fish were examined (Table 1) for the presence of nematodes. All isolated nematodes were morphologically attributable with the species to the genus *Eustrongylides* (Figure 2). Two of them were subjected to molecular analysis, and both showed an identity > 99% with the genus *Eustrongylides.* It was no possible to assign the specimens to the species level because the samples unveiled an identity > 99% with ITS sequences of *Eustrongylides ignotus, Eustrongylides excisus*, and *Eustrongylides* sp. deposited in Genbank.

No visible lesions in external and internal organs were observed. Larvae were found only in the following species: *Lepomis gibbosus*, *Micropterus salmoides*, and *Perca fluviatilis*. All other species tested negative for nematode larvae. The prevalence was 18.3% (15/82) (95% confidence interval (CI) 11–28) in *L. gibbosus*; 16.7% (5/30) (95% CI 6–35) in *M. salmoides*; and 10% (3/30) (95% CI 2–27) in *P. fluviatilis*. No significant differences in prevalence between the three fish species were found (Fisher’s exact test; *p* = 0.744). Table 2 presents the number, location (AV, AD, PV, PD, and VC) of nematode larvae, mean intensity, and mean abundance of infestation. There was a significant difference in larvae localization between muscle quadrants and body cavity (AD = 2; PD = 2; AV = 3; PV = 5; and VC = 19) (Kruskal–Wallis test; *p* = 0.0008), with significant differences between AD and VC (Dunn test; *p* = 0.003), PD and VC (Dunn test; *p* = 0.003), AV and VC (Dunn test; *p* = 0.01), and PV and VC (Dunn test; *p* = 0.03).

## 4. Discussion

Humans are at risk of exposure to parasitic foodborne zoonoses after the consumption of raw or improperly processed fish. The increasing popularity of raw fish consumption has led to a rise in the incidence of parasitic infections [34]. For example, *P. fluviatilis* is an invaluable resource for local fishers. It is largely used in preparing raw and cooked dishes by local people and restaurants. Thus, the presence of *Eustrongylides* nematodes in fish intended for human consumption is potentially harmful for human health.

*Eustrongylides* spp. was previously isolated in three fish species (*Perca fluviatilis, Atherina boyeri*, and *Micropterus salmoides*) from Central Italy [15,18,19]. Our study is the first to report *Eustrongylides* spp. in fish from a subalpine lake (northwest Italy). Also, we found that *Lepomis gibbosus* is a new host for *Eustrongylides* spp. in Italy.

Our morphological and molecular results confirm the specimens as species of the genus *Eustronglyides*. The presence of numerous sequences deposited in GenBank as Eustrongylides sp. could be misleading for further genetic studies, i.e., the construction of phylogenetic tree. This is due to the same similarity value of the ITS sequences between our samples and *Eustrongylides* sp., *E*. *excisus* and *E*. *ignotus* present in GenBank, making difficult the assignment of a nematode to one species rather than another. For this reason, further studies could be focused on other conserved regions.

The prevalence we recorded for *P. fluviatilis* was higher than that reported by fish from central Italy [15,18] (6.84% and 6%, respectively). Also, the prevalence in our *M. salmoides* was higher than the 1.89% reported by Branciari et al. [15]. Other published data on *Eustrongylides* infection in *P. fluviatilis* describes higher prevalence rates than our findings: Goncharov et al. [5] reported a prevalence from 63.6% to 100% (intensity of infestation: 1–13) for different sampling sites in the Ukraine. Sattari [35] reported a prevalence of 33% (mean intensity of infestation, 1.5) in *P. fluviatilis* from Iran. Coyner et al. [36] described the prevalence of *E. ignotus* in 39 fish species from Florida. Moreover, our findings for nematode infestation sites contrast with those reported by other authors [15,18] who found larvae only in skeletal muscle but not in the visceral cavity.

*Eustrongylides* eggs are released by the final hosts throughout the year and may hatch at any time [25]. The eggs remain viable and infective for up to 2 years, and the larvae can survive in the intermediate host for more than 1 year [25]. Piscivorous birds, being a definitive host for numerous parasite species, contribute to their spread [16,37]. The cormorant population has increased in number and area in recent decades in northern Italy [38], where it is concentrated around large lakes and other pre-alpine water basins [38]. Several bird species are well-documented as examples of shifts in geographic distribution related to climate change [39,40]. For parasitic zoonoses, climate change has the potential to shift the boundaries of spatial distribution, host–parasite assemblages, life-cycle phenology, and associations within ecosystems [41]. The microclimate surrounding Lake San Michele and the possibility to nest in protected places such as reed beds make these lentic water bodies favorable for avifauna and for many fish species alike [30].

Lake San Michele has a high concentration of nutrients, typical of eutrophic basins [30]. This may facilitate the growth of oligochaete populations, the intermediate hosts of *Eustrongylides* [36]. *Eustrongylides* larvae have been reported in 14 orders of fish worldwide; the host range is restricted neither to a specific taxonomic group of fish nor to fish with specific feeding habits [42]. A look inside the feeding habits of fish can help us better understand the presence of *Eustrongylides* spp. Largemouth bass, pumpkinseed, and European perch generally inhabit the littoral zone of lakes [43,44,45]. The dimensional and structural attributes of aquatic ecosystems are important determinants of community structure and feeding ecology of fish populations [46]. For example, European perch juveniles feed on zooplankton, bottom invertebrate fauna (i.e., oligochaetes), and other perch fry, while adults feed on both macroinvertebrates and fish [47]. Young largemouth bass also prey mainly on zooplankton, shifting to macrobenthic invertebrates as they grow and then to fish very early [48].

Piscivorous fish may contract multiple infections by consuming infected prey and then host larvae that can survive and remain infective to piscivorous birds [36]. Pumpkinseed feed on zooplankton and benthic invertebrates. Shifts in its diet include replacement of planktonic prey by greater consumption of macroinvertebrates [49]. Frequency of fish consumption by pumpkinseed is reportedly low; thus, it probably acquires *Eustrongylides* by feeding on infected oligochaetes [42]. Each component in the host–parasite relationship is fundamental and determines the dynamics and the outcome of disease transmission and control [50]. Hypothetically, disruption of a parasite’s life cycle may be an effective solution to prevent its spread, but this goal is basically impossible to achieve in natural environments. Further studies are needed to better clarify the link between water quality (i.e., trophic status) and the occurrence of the main oligochaete species involved in the life cycle of nematodes belonging to the *Eustrongylides* genus. Parasite life-cycle stages are distributed across aquatic ecosystem components, and this distribution can vary with place and time. A holistic approach to the management of fishborne parasitic zoonoses entails a large amount of high-quality data and collaboration between the scientific community and public health authorities. 

## 5. Conclusions

Our study reports the prevalence of *Eustrongylides* spp. in a subalpine lake in Italy and improves our knowledge about the distribution and host range of this zoonotic parasite in freshwater environments. *P. fluviatilis* and *M. salmoides* are two fish species of commercial interest and importance for recreational fishing in European lake systems, Italy included [51]. Lake San Michele is popular among sports fishermen but not professional fishermen because of its small size. The presence of these nematodes should be investigated in larger subalpine lakes (i.e., Garda, Como, Maggiore, and Iseo) where commercial fishing is present. While no human infections have been reported in Italy so far, the prevalence of this parasite is possibly underestimated and understudied. Knowledge of numerous aspects of the biology, epidemiology, and control of *Eustrongylides* species is scarce. It is hoped that greater awareness of the zoonotic risk due to changes in dietary habits, globalization through fish product trade, and climatic change will help to inform effective control programs also for fishborne zoonotic parasites.

## Figures and Tables

**Table 1:** Total length (cm) and total weight (g) of fish (N) from Lake San Michele. Plus-minus values are the mean ± standard deviation (SD).

Fish Species	Captured Fish No.	Total Length (cm)	Total Weight (g)
*Ameiurus melas*	30	20.1 ± 2.1	102.3 ± 35.3
*Ictalurus punctatus*	30	26.8 ± 15.1	58.2 ± 28.1
*Squalius cephalus*	30	37.7 ± 5.8	876.9 ± 352.1
*Carassius carassius*	30	34.8 ± 6.2	985 ± 623.7
*Lepomis gibbosus*	82	10.9 ± 2.4	32.6 ± 18.3
*Micropterus salmoides*	30	15.6 ± 3.7	62.2 ± 56.4
*Scardinius erythrophthalmus*	30	21.3 ± 2.1	165 ± 4.5
*Perca fluviatilis*	30	14.3 ± 2.1	42.4 ± 41.4

**Table 2:** Mean intensity and mean abundance of infestation and number and location of larvae in the musculature (anterior ventral (AV), anterior dorsal (AD), posterior ventral (PV), and posterior dorsal (PD)) and in the visceral cavity (VC).

	*Lepomis Gibbosus*	*Micropterus Salmoides*	*Perca Fluviatilis*
Mean intensity	1.15	1	1
Mean abundance	0.28	0.16	0.1
Number of larvae	23	5	3
Location of larvae	2 PD; 5 PV; 16 VC	3 AV; 2 VC	2 AD; 1 VC
